# The Effect of the Substituent Position on the Two-Photon Absorption Performances of Dibenzylideneacetone-Based Isomers

**DOI:** 10.3390/ma9121026

**Published:** 2016-12-20

**Authors:** Liyun Zhao, Yujin Zhang, Hong Ma, Jiancai Leng

**Affiliations:** 1 School of Science, Qilu University of Technology, Jinan 250353, China; liyunzhao_mm@163.com (L.Z.); zhangyujin312@163.com (Y.Z.); 2 School of Physics and Electronics, Shandong Normal University, Jinan 250014, China

**Keywords:** two-photon absorption, optical limiting, dibenzylideneacetone derivatives, isomer, 33.80.-b, 31.15.A-

## Abstract

The two-photon absorption and optical limiting properties of two dibenzylideneacetone derivatives with different substituent positions have been theoretically investigated by solving the coupled rate equations-field intensity equation in the nanosecond time domain using an iterative predictor-corrector finite-difference time-domain method. The calculations show that the electronic structure, the transition dipole moment, the energy gap between the highest occupied orbital (HOMO) and the lowest unoccupied orbital (LUMO), and the pumping rate for the two molecules are quite different due to the different position of chlorine atoms. Importantly, two-photon absorption and optical limiting properties of the molecules depend crucially on the substituent positions of the terminal group, indicating that subtle manipulation on the molecule can affect the nonlinear optical properties of the medium.

## 1. Introduction

Materials with excellent two-photon absorption (TPA) properties have attracted a tremendous amount of interest due to their versatile applications in frequency up-conversion lasing [1,2,3], 3-D microfabrication [4], two-photon fluorescence imaging [5], two-photon photodynamic therapy [6,7], and especially optical limiting (OL) [8,9,10]. Ideal OL materials show stronger nonlinear optical absorption when the intensity of incident laser increases. During the past few decades, with the widespread application of intense lasers as light sources, optical power liming from hazards of the intense laser beam has become an urgent need for instrument protection, eye protection, and optical biology [11]. Therefore, it is an important task for researchers to design and synthesize optical limiters with a large TPA cross section [12,13].

TPA properties of the materials vary in the ability of intermolecular charge transfer, the strength of the donor/acceptor, the character of the conjugated bridge, and the extent of conjugation length [14,15,16,17]. At the same time, previous experimental studies have demonstrated that the substitute position of isomers can affect the distinct nonlinear optical properties [18,19,20]. However, to the best of our knowledge, theoretical investigations on the discrepancy of molecular dynamic TPA properties for isomers are rather insufficient. Most importantly, the consideration of laser-matter interaction on the dynamical nonlinear optical properties of the isomers is still in need, and nonlinear properties of the medium depend strongly on dynamical parameters of the interaction between light and matter, such as the pulse intensity and the propagation distance of the pulse and the density of the medium should be considered.

Dibenzylideneacetone derivatives were reported to possess good TPA properties [21,22]. Furthermore, dibenzylideneacetone-based isomers exhibit different TPA and OL performances experimentally. In this paper, the TPA and OL properties of two dibenzylideneacetone derivatives with different substituent (chlorine group) positions, namely, (1E,4E)-1,5-bis(2-chlorophenyl)-1,4-pentadien-3-one(2-DCDBA) and (1E,4E)-1,5-bis(3-chlorophenyl)-1,4-pentadien-3-one(3-DCDBA) are investigated by solving the coupled rate equations and field intensity equation using an iterative predictor-corrector finite-difference time-domain (FDTD) method under nanosecond regimes. The effect of the substituent position on the nonlinear optical properties of these two derivatives is analyzed. In addition, the dynamical TPA cross sections of the compounds are obtained, and the influence of pulse or medium parameter on the dynamical TPA cross section is compared.

## 2. Theoretical Methods and Computational Details

### 2.1. Rate Equations and Field Intensity Equation

The rate equations for the populations of a three-level system can be written as [23]

(1) $$\begin{array}{c} \frac{\partial }{\partial t}\rho_{s_{0}}=-\gamma_{s_{0}s_{1}}\left(\rho_{s_{0}}-\rho_{s_{1}}\right)-\gamma_{s_{0}s_{2}}\left(\rho_{s_{0}}-\rho_{s_{2}}\right)+\Gamma_{s_{1}}\rho_{s_{1}}\\ \frac{\partial }{\partial t}\rho_{s_{1}}=\gamma_{s_{0}s_{1}}\left(\rho_{s_{0}}-\rho_{s_{1}}\right)-\gamma_{s_{1}s_{2}}\left(\rho_{s_{1}}-\rho_{s_{2}}\right)-\Gamma_{s_{1}}\rho_{s_{1}}+\Gamma_{s_{2}}\rho_{s_{2}}\\ \frac{\partial }{\partial t}\rho_{s_{2}}=\gamma_{s_{0}s_{2}}\left(\rho_{s_{0}}-\rho_{s_{2}}\right)+\gamma_{s_{1}s_{2}}\left(\rho_{s_{1}}-\rho_{s_{2}}\right)-\Gamma_{s_{2}}\rho_{s_{2}} \end{array}$$

where $\Gamma_{s_{n}}$ represents the decay rates of the *S_n_* state, respectively. $\gamma_{S_{0}S_{1}}$ and $\gamma_{S_{1}S_{2}}$ denote the rates of the one photon induced transitions *S*_0_→*S*_1_ and *S*_1_→*S*_2_, which can be given by the one-photon absorption (OPA) cross sections $\sigma_{S_{0}S_{1}}$ and $\sigma_{S_{1}S_{2}}$ or dipole moments $d_{S_{0}S_{1}}$ and $d_{S_{1}S_{2}}$ in the rotating wave approximation (RWA).

(2) $$\begin{array}{c} \gamma_{s_{0}s_{1}}\left(t\right)=\frac{{\left|d_{s_{0}s_{1}}\right|}^{2}I\left(t\right)}{\hbar^{2}c\varepsilon_{0}}\frac{\Gamma }{{Ω}_{s_{0}s_{1}}^{2}+\Gamma^{2}}=\frac{\sigma_{s_{0}s_{1}}I\left(t\right)}{\hbar \omega }\frac{\Gamma^{2}}{{Ω}_{s_{0}s_{1}}^{2}+\Gamma^{2}}\\ \gamma_{s_{1}s_{2}}\left(t\right)=\frac{{\left|d_{s_{1}s_{2}}\right|}^{2}I\left(t\right)}{\hbar^{2}c\varepsilon_{0}}\frac{\Gamma }{{Ω}_{s_{1}s_{2}}^{2}+\Gamma^{2}}=\frac{\sigma_{s_{1}s_{2}}I\left(t\right)}{\hbar \omega }\frac{\Gamma^{2}}{{Ω}_{s_{1}s_{2}}^{2}+\Gamma^{2}}\\ {Ω}_{s_{0}s_{1}}=\omega -\omega_{s_{0}s_{1}},\;{Ω}_{s_{1}s_{2}}=\omega -\omega_{s_{1}s_{2}} \end{array}$$

Similarly, the rate of two-photon induced transition *S*_0_→*S*_2_ can be calculated by the TPA absorption cross section $\sigma_{S_{0}S_{2}}$:

(3) $$\gamma_{s_{0}s_{2}}\left(t\right)=\frac{\sigma_{s_{0}s_{2}}I^{2}\left(t\right)}{2\hbar \omega }\frac{\Gamma^{2}}{{\left(2\omega -\omega_{s_{0}s_{2}}\right)}^{2}+\Gamma^{2}}$$

where ω is the light frequency, and *I*(*t*) is the intensity of the incident light field. Γ denotes the homogeneous broadening of the spectral line, which is set as ℏΓ = 0.1 eV in our simulation [24].

As regards the electromagnetic field, the absorption of the field can be described by using the field intensity *I*(*t*) [22]:

(4) $$\left(\frac{\partial }{\partial z}+\frac{1}{c}\frac{\partial }{\partial t}\right)I\left(t\right)=-N\left[\sigma^{\left(1\right)}I\left(t\right)+\sigma^{\left(2\right)}I^{2}\left(t\right)\right]$$

where *N* is the molecular density. σ^(1)^ and σ^(2)^ are the total OPA and TPA cross sections, respectively, and they can be expressed as

(5) $$\sigma^{\left(1\right)}=\sigma_{s_{0}s_{1}}\left(\rho_{s_{0}}-\rho_{s_{1}}\right)+\sigma_{s_{0}s_{1}}\left(\rho_{s_{1}}-\rho_{s_{2}}\right)\;\sigma^{\left(2\right)}=\sigma_{s_{0}s_{2}}\left(\rho_{s_{0}}-\rho_{s_{2}}\right)$$

### 2.2. Static TPA Cross Section

Considering the transition characteristics of the molecule, the static maximum TPA cross section of the three-level system can be expressed as

(6) $$\sigma_{STPA}\propto \frac{{d^{2}}_{s_{0}s_{1}}{d^{2}}_{s_{1}s_{2}}}{{\left(E_{s_{0}s_{1}}-E_{s_{0}s_{2}}/2\right)}^{2}\Gamma_{f}}+\frac{{d^{2}}_{s_{0}s_{2}}{\left(d_{s_{2}s_{2}}-d_{s_{0}s_{0}}\right)}^{2}}{{\left(E_{s_{0}s_{2}}/2\right)}^{2}\Gamma_{f}}$$

where *d*_mn_ is the permanent dipole moment of state, and *E*_mn_ is the excitation energy. The final level broadening definition $\Gamma_{f}$ defined as a common value of 0.1 eV. For the one-dimensional symmetrical structure of the molecule, the permanent dipole moments of the molecule are approximately equal to zero. Thus a simplified form can be obtained:

(7) $$\sigma_{STPA}\propto \frac{{d^{2}}_{s_{0}s_{1}}{d^{2}}_{s_{1}s_{2}}}{{\left(E_{s_{0}s_{1}}-E_{s_{0}s_{2}}/2\right)}^{2}\Gamma_{f}}$$

### 2.3. Dynamical TPA Cross Section

Taking into account that the TPA coefficient β is related to the incident light intensity, we use a linear approximation to represent the TPA coefficient [23,24]:

β = β_0_ − ξ*I*_0_ (8)

where β_0_ is the static state TPA coefficient, and ξ is a constant. The reciprocal of the light intensity transmittance can be expressed as quadratic function of the input field intensity *I*_0_.

(9) $$\frac{1}{T_{z}}=\frac{I_{0}}{I_{z}}=\exp\left(az\right)+\frac{\left[\exp\left(az\right)-1\right]\beta }{\alpha }I_{0}-\frac{\left[\exp\left(az\right)-1\right]\xi }{\alpha }I_{0}^{2}$$

where α is the linear absorption coefficient. The molecular TPA cross section σ_tp_ is related to β by

(10) $$h\nu \beta =\sigma_{\mathrm{tp}}N$$

where *hν* is the incident photon energy. Therefore, we can obtain the dynamical TPA cross section by fitting the absorption coefficients.

## 3. Results and Discussion

**Figure 1:**
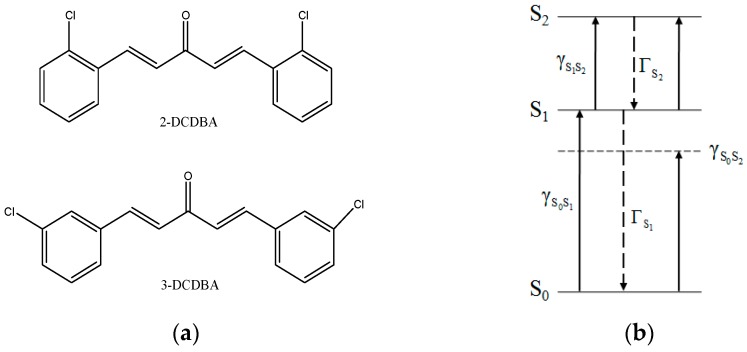
(**a**) Molecular structure diagrams of 2-DCDBA and 3-DCDBA; (**b**) scheme of the three-level theory model.

Molecular structures of 2-DCDBA and 3-DCDBA are shown in Figure 1. The excitation energies of the excited states in the low energy region and transition dipole moments between the energy levels are calculated by using the time-dependent density functional theory(TDDFT) at the B3LYP/6-31G(d) level. The corresponding values are collected in Table 1. It is shown that both molecules can be simplified to a three-level system, including the ground state *S*_0_, the charge-transfer state *S*_1_, and the TPA state *S*_2_, as shown in Figure 1b. From state *S*_0_ to state *S*_1_ for both molecules, transition dipole moments differ by 0.4 × 10^−30^ C∙m, while the state *S*_1_ to state *S*_2_, the transition dipole moment differs by 0.31 × 10^−29^ C∙m.

The incident pulse is modeled with a hyperbolic secant shape:

(11) $$E\left(z,t=0\right)=A_{0}\mathrm{sech}\left[1.76\left(z/c+z_{0}/c\right)/\tau \right]\cos\left[\omega \left(z+z_{0}/c\right)\right]$$

where A_0_ is the peak amplitude of the input field, and τ is the full width at half maximum (FWHM) of the pulse intensity profile which is set to be 8ns in our calculation. The selection of z_0_ is to ensure that initial pulse into the medium is seldom at *t* = 0. In order to demonstrate the TPA behaviors, the frequency of the pulse ω is taken to be half of the two photon resonance frequency between states *S*_0_ and *S*_2_, namely, $\omega =\omega_{s_{0}s_{2}}/2$. The decay rates of excited states $\Gamma_{S_{1}}$ and $\Gamma_{S_{2}}$ are assumed to be 1.0 × 10^9^/s, 1.0 × 10^12^/s, respectively [25]. The initial peak intensity is set as 1 × 10^8^ W/m^2^. Before the laser pulse incident into the medium, all of the molecules are at ground state, that is, $\rho_{S_{0}}\left(t=0\right)=1$, $\rho_{S_{1}}\left(t=0\right)=\rho_{S_{2}}\left(t=0\right)=0$.

**Figure 2:**
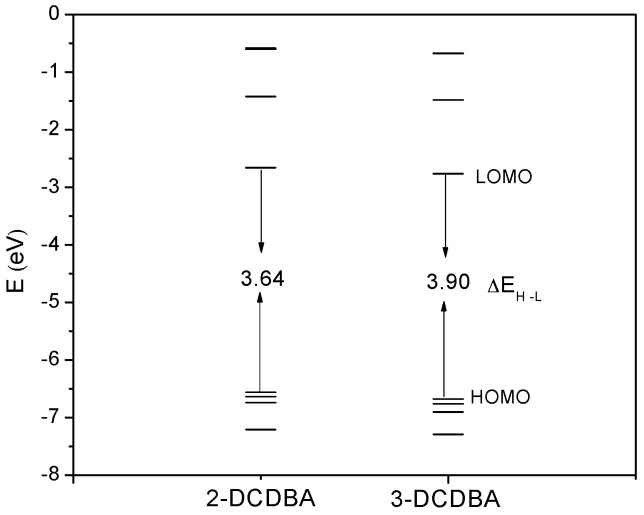
Molecular frontier orbital energy level diagram for 2-DCDBA and 3-DCDBA.

The frontier orbitals and the energy gap between the highest occupied orbital (HOMO) and the lowest unoccupied orbital (LUMO) of 2-DCDBA and 3-DCDBA are depicted in Figure 2. It can be seen that the energy gap of 3-DCDBA (3.90 eV) is larger than that of 2-DCDBA (3.64 eV), which originates from the change in the substituent group position.

**Figure 3:**
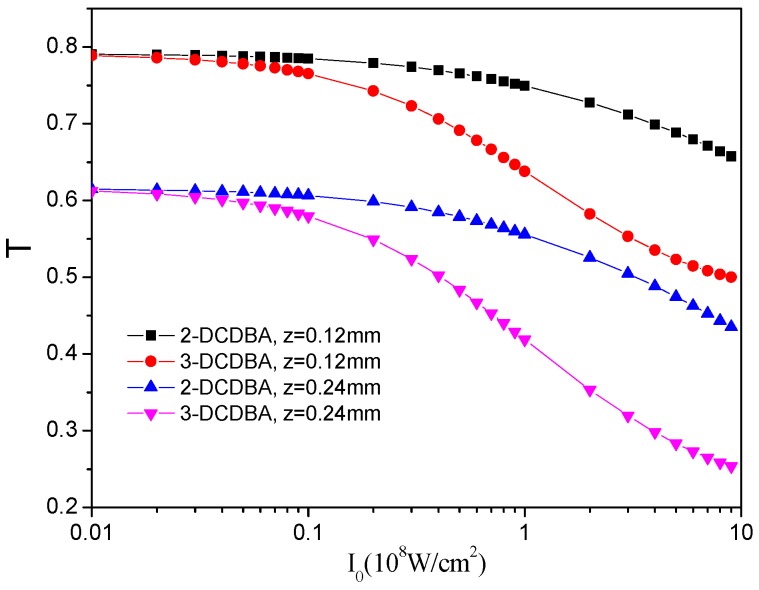
Field intensity transmittance as a function of the 2-DCDBA and 3-DCDBA incent light field at different propagation distances.

To explore the TPA properties of the two molecules, we give the transmittances of incent light field intensity for 2-DCDBA and 3-DCDBA at different propagation distances in Figure 3. It is evident that both mediums exhibit excellent nonlinear absorption ability. The transmittance is almost constant at low incident light intensity, suggesting that linear absorption plays a dominant role in this region. However, transmittance decreases rapidly with the increase in incident light intensity, which originates from the TPA process in the three-level system. It is shown in Figure 3 that, for a certain molecule, the transmittance is smaller for a longer propagation distance because more energy of the field is transferred into the medium as pulse propagation. Obviously, transmittance for 3-DCDBA is much lower than that of 2-DCDBA, which means 3-DCDBA is preferred as an absorber. This result indicate that different positions of the chlorine substituent group lead to an influence on intensity transmittances, and the meta position is better than the ortho position. 

**Figure 4:**
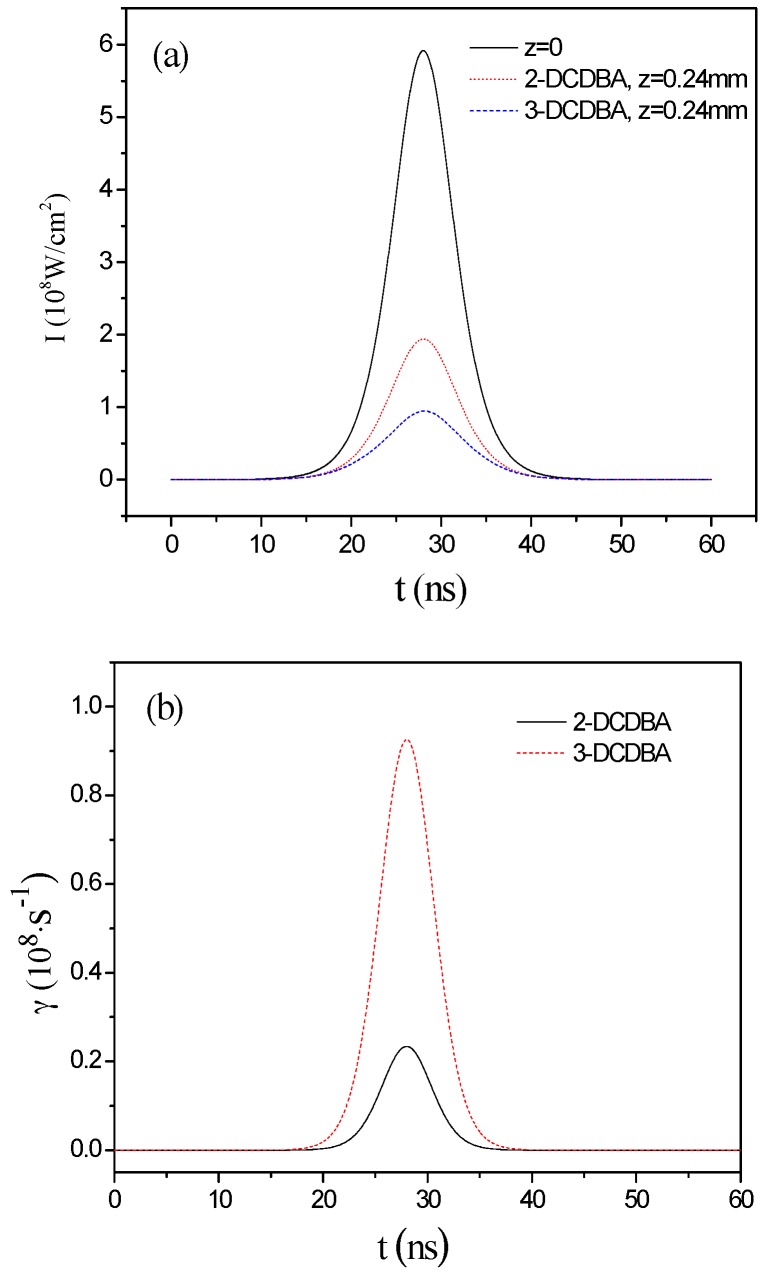

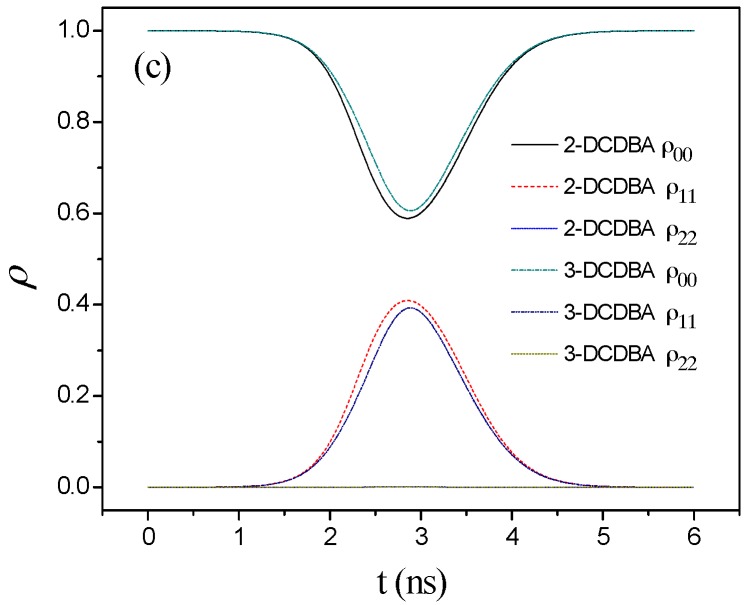
(**a**) The changes in the pulse intensity envelope; (**b**) the pump rate (*I* = 6 × 10^8^ W/cm^2^, *z* = 0.24 mm); and (**c**) the particle number density of three levels for 2-DCDBA and 3-DCDBA (*I* = 6 × 10^8^ W/cm^2^, *z* = 0.24 mm).

When the laser pulse propagates in the medium, the pulse strength envelope, the pump rate, and the particle number density of three levels are changed, as demonstrated in Figure 4. Figure 4a shows that pulse intensities are decreased in relation to pulse propagate process due to the interaction between the field and the medium, and the energy of the field are transferred into molecules. In addition, as shown in the figure, more energy is transferred for 3-DCDBA than that for 2-DCDBA during pulse propagates, which results from the substituent position. Figure 4b displays the pump rate of the TPA process for the molecules with a pulse intensity of 6 × 10^8^ W/cm^2^ at *z* = 0.24 mm. It is shown that the TPA rate of 3-DCDBA is much larger than that of 2-DCDBA, indicating that 2-DCDBA needs more time to become excited to a higher state. Figure 4c shows that most of the molecules are excited to the OPA state *S*_1_, while the TPA state *S*_2_ is almost unpopulated. This phenomenon is understandable: the lifetime of state *S*_2_ is on the picosecond scale, and populations on state *S*_2_ will decay rapidly back to the state *S*_1_.

**Figure 5:**
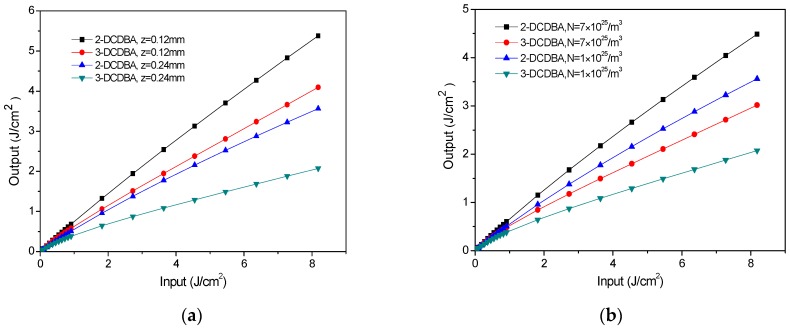
Optical limiting behaviors of 2-DCDBA and 3-DCDBA at (**a**) different propagation distances (*N* = 1 × 10^25^/m^3^) and (**b**) different particle number densities (*z* = 0.24 mm).

The curves of output fluence versus input fluence for both mediums at different propagation distances and particle number densities are shown in Figure 5. One can see that input fluence significantly decrease with the increase in propagation distances and particle number densities. It is obvious that, with the increase in the medium length and particle number densities, more energy of the field is absorbed due to the interaction between the field and the medium. This suggests that the OL performance is more apparent for longer propagation distance and larger particle number densities, leading to an enhancement in the OL abilities. Consistent with previous conclusions, the OL performance of 3-DCDBA is much stronger than that of 2-DCDBA. 

Based on the input–output peak intensity relation, we obtained dynamical TPA cross sections of 2-DCDBA and 3-DCDBA at propagation distances of 0.12 mm and 0.24 mm, as shown in Table 2. Calculation results show that the values of the TPA coefficient β and the dynamical TPA cross section σ_tp_ for both molecules increase with the increase in propagation distances, and the TPA cross section of 3-DCDBA is larger than the 2-DCDBA at different propagation distances. Therefore, the values of the TPA cross section depend crucially on the thickness of the medium. Compared with the static TPA cross sections of 2-DCDBA and 3-DCDBA, which are 356 GM (1 GM = 10^−50^ cm^4^s/photon) and 396 GM, respectively, the dynamical TPA cross sections are two orders of magnitude larger. This is because the dynamical TPA cross section has taken the two-step TPA process into consideration, while the static TPA cross section only includes the contribution of the one-step TPA. In the case of both static and dynamical conclusions, 3-DCDBA has a larger TPA cross section compared with 2-DCDBA, showing that the nonlinear absorption performance of 3-DCDBA is better than that of 2-DCDBA due to the different position of the substituent group.

## 4. Conclusions

Dynamical analysis of optical limiting and TPA activities of two dibenzylideneacetone derivatives 2-DCDBA and 3-DCDBA were studied using the dynamical nonlinear absorption theory of nanosecond laser pulses. Our numerical results show that electronic structures, transition dipole moments, HOMO–LUMO energy gaps, and pump rates are influenced by the position of the substituent group, and 3-DCDBA, with the position of the substituent group at the meta position, has preferable nonlinear absorption properties, with a change in propagation distance and particle number density of the medium. This indicates that TPA and OL properties of the molecules depend crucially on the substituent positions of the terminal group, and subtle manipulation on the molecule can affect the nonlinear optical properties of the medium. Our theoretical results explain that the difference in nonlinear optical properties for the isomers and provide insight on the design of organic molecules with good TPA properties.

## Figures and Tables

**Table 1:** Excitation energies and transition dipole moments of 2-DCDBA and 3-DCDBA.

Molecule	$E_{s_{0}s_{1}}/\mathrm{eV}$	$E_{s_{1}s_{2}}/\mathrm{eV}$	$d_{s_{0}s_{1}}/(10^{-29}\;C\cdot m$)	$d_{s_{1}s_{2}}/(10^{-29}\;C\cdot m$)
2-DCDBA	3.51	3.80	2.85	4.38
3-DCDBA	3.55	3.83	2.89	4.69

**Table 2:** The values of linear absorption coefficient α (10^3^ m/W), the TPA coefficient β (nm/W), dynamical TPA cross section σ_tp_ (10^5^ GM), and the static TPA cross section (GM) of 2-DCDBA and 3-DCDBA at different propagation distances.

Molecule	*z*	α	β_0_	σ_tp_	σ_STPA_
2-DCDBA	0.12 mm	1.98	0.40	1.23	366
	0.24 mm	1.04	0.81	2.48	
3-DCDBA	0.12 mm	4.09	0.89	2.72	396
	0.24 mm	2.22	1.92	5.90	
